# The Patriotism of the Nurse-Training Schools

**Published:** 1917-03-31

**Authors:** 


### THE PATRIOTISM OF THE NURSE-TRAINING SCHOOLS.

### XII. London Training Schools.

It is not easy to catalogue the sisters and nurses who have been dispatched from the major training-schools to the seat of war. The mere mention of figures suffices in the case of St. Bartholomew's to convey a vivid picture of the reorganisation undertaken by the authorities in the interests of the wounded. We are able to give a complete list of the members of the Westminster staff who have been set free for war duty, and are glad to record their names.

### St. Bartholomew's Hospital.

No list of nurses furnished from the hospital to the Navy and Army Nursing Services is available. But some interesting particulars relating to the numbers sent out and their services are appended.

Since 1910 St. Bartholomew's has annually renewed undertakings given to the Admiralty and to the War Office to supply twenty-eight certificated nurses for the Navy and thirty for the Army, in the event of war or national emergency. On August 8, 1914, in response to the request of the Matron-in-Chief of Queen Alexandra's Imperial Military Nursing Service, the first party of three nurses was sent. Others were asked for at frequent intervals, and by September 15, 1914, the number originally promised had been completed. With the exception of five, all these nurses were detailed to hospitals in France or to hospital ships, and those five nurses who were serving in England and Ireland at military hospitals soon joined their colleagues in France. Since that date twenty-eight more nurses have been supplied for France, two for Gibraltar, twenty-six for Malta, three for Salonica, and seven for Egypt, all on the Civil Reserve.

In addition to these nurses, twenty-five certificated nurses left the service of the hospital and the private nursing staff to join the Territorial unit at the 1st London (City of London) General Hospital at Camberwell, and the certificated staff of that hospital was until recently entirely composed of nurses belonging to St. Bartholomew's Training School.

Many of the nurses of this hospital joined on their own account the Q.A.I.M.N.S.R., and are serving in different capacities in military hospitals at home and abroad. Others have joined various nursing organisations in connection with the war. In fact there are not less than 263 nurses trained at this hospital who are serving in the Navy, Army, and Territorial Forces.

Thirteen nurses holding St. Bartholomew's Hospital certificate and now belonging to Q.A.I.M.N.S. and other organisations connected with the war have been awarded the Royal Red Cross (1st class). Twelve others have been awarded the 2nd class Order of the Royal Red Cross, and twenty-two have been mentioned in dispatches.

Of the twenty-eight nurses promised by the governors of the hospital for Q.A.R.N.N.S.R., seven nurses were called up on August 4, 1914, and these were followed at intervals by others, the total number supplied up to February 1917 being seventeen. Three of these have been detailed to the Naval Hospital at Malta, and one to H.M.S. *China*. The remainder are serving in naval hospitals in England.

Between August 1914 and May 1915 the matron took, in relays, 108 members of the St. John Ambulance Association and the British Red Cross for three weeks' practical experience in the wards. Since then three months' training has been granted to eighty-three probationers, the majority of whom are now working in military hospitals.

### Westminster Hospital.

Many nurses from the Westminster Training School have been engaged in war service since the commencement of the war, and the number is now over sixty. We append some of the names which have been supplied to us:—

Sisters Chibnall, Cooper, Ford, and Edwards; military hospitals at home. Nurses Vickers, Bourne, Haywood, Anscombe, Knight, Rumbal, Blainire Brown, Macgillivory, Soutar, Sybil Grey, Shepherd Smith, Haggerson, Pearson, Armitage, Brebner, Houston, McLean, Howarth, and Amy Nicholls; military hospitals at home. Nurses Riddlesdell, Rushworth, Silkstone, Gover, Atkins, Combes, Barratt, Cutcliffe, Peacock, Taylor, and Smith; British Expeditionary Force in France. Nurses Burnett, Keys, Hudson, and Robertson; Mediterranean. Nurses Vernon and F. Jackson to Egypt; Nurses Douglas and Hewson to Boulogne; Nurses Dorothy Lewis and Salmond to Netley; Nurses Warren and Cayless to R.N. Hospital, Chatham. Nurses Howson, Robertson, and Banton have been sent to Malta; Nurses Allan and Chace to Salonika.

Miss Maud Knight, trained at this hospital, has been mentioned in dispatches. Sister Ford and Sister Farrington have been honoured by the bestowal of the Royal Red Cross (2nd class). Miss Hilda Chibnall, who has been serving on a hospital ship, has been recommended for the Royal Red Cross (2nd class).

One hundred beds in Westminster Hospital are at the disposal of the War Office, and there are always between ninety and a hundred soldier-patients in residence, so that many of the nursing staff are doing war nursing in their own hospital.

